# The economic burden of perinatal mortality due to inaction on preconception health in low and middle-income countries: A population attributable fraction and economic impact analysis

**DOI:** 10.1371/journal.pone.0325086

**Published:** 2025-07-03

**Authors:** Sébastien Poix, Patrick O’Donnell, Khalifa Elmusharaf

**Affiliations:** 1 Faculty of Education & Health Sciences, School of Medicine, University of Limerick, Limerick, Ireland; 2 Public Health, Applied Health Research, University of Birmingham Dubai, Dubai, United Arab Emirates; Public Library of Science, UNITED KINGDOM OF GREAT BRITAIN AND NORTHERN IRELAND

## Abstract

**Background:**

Poor preconception health has been associated with several pregnancy and childbirth-related complications, including perinatal mortality. Yet, the health and economic burden that inaction on preconception health places on societies remains under-researched, hindering efforts to address these issues effectively. This study aimed to quantify the economic burden of perinatal mortality attributable to five preconception risk factors in fifteen low and middle-income countries (LMICs).

**Methods:**

We used a population-attributable fraction analysis to estimate the proportion of perinatal deaths in 2020 attributable to adolescent pregnancy, short birth intervals, intimate partner violence before pregnancy, pre-pregnancy overweight and obesity, and female genital mutilation. We then performed an economic impact analysis to quantify the foregone productivity and the societal costs due to these perinatal deaths, using both the human capital and the value of a statistical life-year approach.

**Findings:**

More than 230,000 (20.7%) perinatal deaths were attributable to the five selected risk factors in the fifteen LMICs in 2020. The productivity losses were estimated at $INT21.3 billion, representing 0.7% of the combined GDP of those countries in 2020. The societal costs of perinatal mortality, the total economic burden was $INT51.0 billion.

**Interpretation:**

Our findings indicate that inaction on preconception care potentially contributes to a substantial proportion of the burden of perinatal mortality, which, in turn, generates profound and long-term economic and societal losses in LMICs. These results highlight the need for effective preconception strategies and relevant policies, and further research is needed to explore the economic value of preconception care in these settings.

## Introduction

Perinatal mortality, which encompasses deaths occurring during the period from twenty-two completed weeks of gestation to seven days after birth [1], remains a significant public health issue globally. Approximately 2.3 million newborn children died in 2022, making the neonatal period the most precarious for child survival [2]. The burden of neonatal mortality is disproportionately borne by low and middle-income countries (LMICs), notably in Sub-Saharan Africa and Central and Southern Asia. Despite the substantial progress that has been made since 2000, many LMICs will fall behind the Sustainable Development Goal (SDG) target of reducing neonatal mortality to at least 12 per 1,000 live births by 2030 [3]. Stillbirths represent a significant aspect of perinatal mortality, with nearly 2 million occurring around the world every year [4]. In 2014, the World Health Assembly endorsed the Every Newborn Action Plan (ENAP), which set a global target of 12 or fewer late stillbirths per 1,000 total births by 2030 [5]. However, current trends indicate that more than 50 countries, mainly LMICs, will not reach this target without accelerated action [4].

Effective strategies to reduce the burden of perinatal mortality include the provision of timely, regular, and good-quality antenatal care, accessible emergency obstetric care, birth attendance by trained health professionals, and immediate and high-quality care for newborns, including resuscitation and infection management. Additionally, educating mothers and families on maternal and newborn health plays a significant role in preventing perinatal deaths [5].

In the last decade, the promotion of preconception health has been highlighted as another way to reduce pregnancy-related complications and child morbidity and mortality [6–8]. This increased focus builds on the idea that various risk factors associated with adverse pregnancy, maternal, and child outcomes are rooted within the preconception period. By addressing these risk factors and ensuring better pregnancy readiness, women can embark on pregnancy in optimal health, ultimately leading to improved foetal development, reduced complications, and long-term well-being for both mothers and children [9]. The World Health Organization (WHO) identified many intervention areas, including but not limited to malnutrition, harmful substance use, early and rapid successive pregnancies, interpersonal violence, female genital mutilation, mental health disorders, or exposure to environmental risks [10].

In 2012, as part of a meeting to develop a global consensus on the topic, WHO recognised the need to integrate preconception care into the continuum of care that extends from pregnancy to the postnatal period [11]. However, global progress in developing comprehensive and integrated preconception strategies and policies has been limited since then. Enhanced advocacy is needed to foster better integration of preconception care into existing healthcare frameworks. Building the case for governments to increase their investment in preconception health can significantly support these efforts.

Assessing the cost and economic benefits of investing in preconception health is essential to garnering the needed political and societal support, informing decision-making, and guiding effective resource allocation. A preliminary step to such analysis is to better understand the health and economic burden that inaction on preconception health places on societies. Perinatal mortality is one of the main outcomes associated with poor preconception health. By decreasing future spending on goods and services, reducing tax revenues, and diminishing the potential labour force, perinatal deaths negatively affect future productivity and weaken the economy’s capacity to grow and develop [12,13].

This study estimates the economic burden of perinatal mortality due to inaction on five preconception risk factors in a selection of fifteen LMICs using 2020 data. It has two aims. The first is to measure the number of perinatal deaths attributable to adolescent pregnancy, short birth spacing, pre-pregnancy overweight and obesity, intimate partner violence, and female genital mutilation. The second is to estimate the productivity and societal costs of these perinatal deaths. By employing this two-step methodology, we assessed the costs that could theoretically be averted if these preconception risk factors were eliminated.

## Materials and methods

### Approach

**Fig 1:**
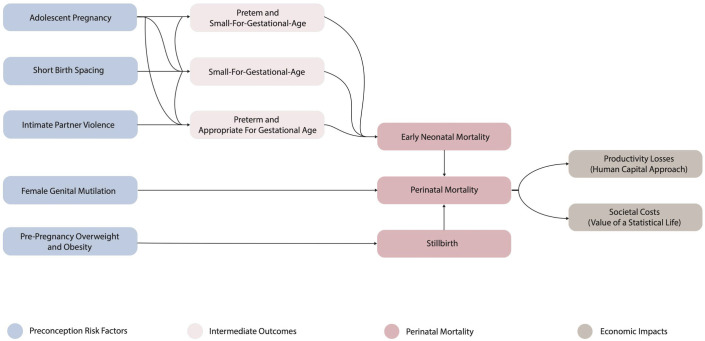
Conceptual framework.

We combined a population-attributable fraction analysis and an economic impact analysis to estimate the productivity losses and the societal costs of perinatal mortality attributable to adolescent pregnancy, short birth spacing, pre-pregnancy overweight and obesity, intimate partner violence before pregnancy, and female genital mutilation in a selection of fifteen countries in 2020 (Fig 1). While these preconception risk factors have been associated with various adverse pregnancy-related outcomes (i.e., low birth weight, postpartum haemorrhage, etc.), we focused on perinatal mortality because evidence linking these five risk factors to this outcome is both consistent and robust. We estimated the foregone productivity using a human capital approach, whilst a value of a statistical life-year approach was utilised to model the societal costs of perinatal mortality. To allow comparisons between countries, all the costs are provided in 2020 INT$, using purchasing power parity (PPP) conversion factors. Future costs were discounted at 3%, a standard rate in global health economic analyses [14]. This process reflects the time value of money, capturing the principle that one dollar today is worth more than a dollar in the future.

### Guidelines

We followed the Guidelines for Accurate and Transparent Health Estimates Reporting [15] to guide the reporting of our methodology and ensure transparency and reproducibility of our analysis (S1 Appendix).

### Selected countries

The productivity and societal costs of perinatal deaths attributable to the selected preconception risk factors were estimated for Afghanistan, Burundi, Benin, Chad, Côte d’Ivoire, Guinea, Gambia, Kenya, Liberia, Lesotho, Mali, Mauritania, Nigeria, Pakistan, and Sierra Leone. We used two inclusion criteria to select these countries. First, the neonatal mortality rate exceeded 30 neonatal deaths per 1,000 live births. Second, a Demographic Health Survey (DHS) had to be published in the country after 2014 [16]. DHS country data were used as the primary source of information to determine the prevalence of short birth spacing, overweight and obesity, intimate partner violence and female genital mutilation, and this was to ensure that relevant data were available and were sufficiently recent.

### Population attributable fraction analysis

We employed a five-step methodology to estimate the number of perinatal deaths attributable to the selected preconception risk factors. First, we determined the prevalence of the preconception risk factors based on available evidence. The prevalence of adolescent pregnancy in primiparous women was determined by combining data from the UN World Population Prospects [17] and the DHS [16]. The prevalence of short birth spacing, overweight and obesity, intimate partner violence, and female genital mutilation was obtained from the DHS. Intimate partner violence was defined as sexual and physical violence committed by the husband or partner in the 12 months preceding the survey. We only considered the female genital mutilation of type II and III, whose association with perinatal mortality is supported by robust evidence. According to WHO’s classification, female genital mutilation type II is defined by the excision of the clitoris with partial or total removal of the labia minora. In contrast, female genital mutilation type III refers to the excision of part or all of the external genitalia and stitching or narrowing of the vaginal opening [18]. The prevalence of female genital mutilation was not available in Afghanistan, Burundi, Pakistan, and Lesotho, either because it was too low, it was not collected, or it was not reported. Therefore, the perinatal deaths attributable to female genital mutilations of type II and III were calculated for the eleven other countries only. We used prevalence rates disaggregated by five-year age intervals to capture a maximum nuance in the results (S1 Appendix).

Second, the relative risks (RRs) measuring the association between the risk factors and perinatal mortality were derived from five meta-analyses and one prospective study conducted in Ghana, Burkina Faso, Nigeria, Kenya, Senegal, and Sudan [18–23] (S1 Appendix). We considered RRs of perinatal mortality, stillbirths or early neonatal deaths. For adolescent pregnancy, short birth spacing, and intimate partner violence, we used RRs for preterm birth (PTB-AGA), preterm birth with small-for-gestational-age (PTB-SGA), and small-for-gestational-age (SGA) as intermediate outcomes. In two meta-analyses, the risk relationships were expressed in odd ratios (ORs). We converted these ORs into RRs using the methodology described by Zhang and Yu [24] (S1 Appendix). We used RR estimates adjusted for potential confounding factors, except for the relationship between intimate partner violence before pregnancy and preterm birth. In this case, the authors reported unadjusted data due to a lack of available evidence [22].

Third, we estimated the population-attributable fraction (PAF) of perinatal deaths due to the preconception risk factors. The PAF represents the proportion of perinatal deaths that could theoretically be prevented if all five preconception risk factors were eliminated. We calculated it using Levin’s formula [25]:


$${\text{PAF}}_{\text{u}}=\frac{{\text{P}}_{\text{e}}\times (\text{RR}-1)}{{\text{P}}_{\text{e}}\times (\text{RR}-1)+1}$$

Where P_e_ is the prevalence of the risk factor, and RR represents the relative risk of a health outcome in the population exposed to the risk factor compared to those not exposed. As mentioned above, we calculated the PAF of perinatal deaths due to adolescent pregnancy, short birth spacing and intimate partner violence in two stages. Firstly, we estimated the PAF of PTB-AGA, PTB-SGA and SGA due to these risk factors. Then, we calculated the PAF of perinatal deaths due to cases of PTB-AGA, PTB-SGA and SGA attributable to the three preconception risk factors. Following the methodology described by Bryce et al. [26], we used additional formulas to ensure that estimates of the PAF accounted for the interactions and combined effects of multiple risk factors (e.g., adolescent pregnancy and intimate pregnancy). The detailed methodology is presented in the supplementary information (S1 Appendix).

Fourth, we calculated the total cases of perinatal deaths by adding the cases of stillbirths and early neonatal deaths. The cases of stillbirths were estimated by combining fertility indicators from the UN World Population Prospects and stillbirth rates from the WHO Global Health Observatory [27]. To estimate the number of early neonatal rates, we used the neonatal mortality rate from the WHO Global Health Observatory and the proportion of neonatal deaths occurring in the first seven days after childbirth derived from the Institute of Health Metrics Global Burden of Disease 2019 [28].

Fifth, we calculated the number of perinatal deaths attributable to adolescent pregnancy, short birth spacing, pre-pregnancy overweight and obesity, intimate partner violence before pregnancy and female genital mutilation by multiplying the total number of perinatal deaths by the corresponding PAFs.

### Economic impact analysis

We employed two distinct methodologies to assess the economic impact of perinatal mortality attributable to the selected preconception risk factors: (1) the human capital approach and (2) the value of a statistic life-year (VSLY) approach. The human capital approach quantifies the foregone productivity due to the premature deaths of individuals who would have participated in the workforce had they survived. This method estimates tangible economic losses, emphasising the direct impact of perinatal mortality on national productivity. The VSLY approach builds on society’s willingness to pay for reducing mortality risks to estimate the value of every additional year of life. Unlike methods that focus solely on lost productivity, the VSLY captures the broader societal cost of mortality by valuing life itself. This approach provides a more comprehensive assessment of the economic burden, accounting for both monetary and non-monetary impacts of perinatal mortality.

We used the following formula to estimate the future productivity losses due to perinatal mortality [29]:


$$D\times L\times E\times \left[\sum {\mathrm{\backslash nolimits}}_{t=S}^{R}GDPW/(1+r{)}^{t-S}\right]$$

where D is the perinatal deaths attributable to the preconception risk factors, L is the labour force participation rate, E is the employment rate, S is the age when individuals would have started working, R is the retirement age, GDPW is the gross domestic product (GDP) per working-age population, and r is the discount rate. We obtained the labour force participation and the employment rate from the International Labour Organisation database (ILOSTAT) [30]. To calculate the GDP per working-age population, we sourced the GDP from the World Bank online database [31] and divided it by the working-age population. We assumed the minimum working age was 15 and the retirement age was 64 in all the selected countries (S1 Appendix).

We calculated the societal costs of perinatal mortality using the formula as follows [32]:


$$D\times \left[\sum {\mathrm{\backslash nolimits}}_{t=0}^{LE}VSLY/(1+r{)}^{t}\right]$$

where D is the perinatal deaths attributable to the preconception risk factors, LE is the life expectancy at birth, VSLY is the income-adjusted value of a statistical life-year, and r is the discount rate. The life expectancy at birth was obtained from the WHO Global Health Observatory [27]. Following the methodology described by Viscusi and Masterman [33], we estimated the value of a statistical life (VSL) in each country by transferring it from an estimate of the VSL in the United States in 2020 [34]. The transfer was realised using the GNI per capita from the World Bank database [31] and an income elasticity of 1.103. We then annualised the VSL by dividing it by the life expectancy of an individual of median age in each country, which we obtained from the UN World Population Prospects [17] and the WHO Global Health Observatory [27] (S1 Appendix).

### Sensitivity analysis

Because PAF estimates are highly sensitive to RRs, we conducted the analysis using the lower and upper bounds of the confidence intervals of the RRs. We also explored variations in the results when using a discount rate of 5% and 7% [35].

## Results

In 2020, there were 21,503,945 births reported in the fifteen countries, with 65.5% of these occurring in Pakistan and Nigeria. The total number of perinatal deaths was estimated at 1,125,296, which represents an average rate of 53.7 perinatal deaths per 1,000 live births. The average GDP per working-age population is $INT5,699 (SD = $INT2,779), with substantial variations from $INT1,476 in Burundi to $INT10,151 in Mauritania. The average income-adjusted VSL is $INT437,051 (SD = $INT237,882). It ranges from $INT93,605 in Burundi to $INT809,889 in Côte d’Ivoire and Mauritania (S1 Appendix).

We estimated that 20.7% of all perinatal deaths in the fifteen selected countries were attributable to adolescent pregnancies, short birth spacing, pre-pregnancy overweight and obesity, intimate partner violence before pregnancy, and female genital mutilation in 2020 (Table 1). Sierra Leone had the largest PAF (34.8%), followed by Guinea (32.2%) and Mauritania (31.7%). The smallest PAFs were found in Burundi (7.5%) and Lesotho (10.6%), two countries in which female genital mutilation is officially not practised. When taking into consideration the countries for which the impact of female genital mutilation was modelled, the smallest PAF was observed in Benin (12.5%). Overall, we estimated that 233,347 perinatal deaths were attributable to the selected preconception risk factors in 2020, including 61,029 in Nigeria and 98,080 in Pakistan.

**Table 1:** Proportion attributable fraction (PAF) of perinatal deaths for the selected preconception risk factors in 2020.

Country	Total perinatal deaths	Perinatal deaths attributable to the preconception risk factors	PAF (%)
Afghanistan*	71,621	15,972	22.3%
Burundi*	14,747	1,100	7.5%
Benin	20,875	2,617	12.5%
Côte d’Ivoire	44,313	10,765	24.3%
Guinea	21,278	6,841	32.2%
Gambia	3,774	1,212	32.1%
Kenya	49,127	8,101	16.5%
Liberia	7,490	1,767	23.6%
Lesotho*	2,785	294	10.6%
Mali	44,791	12,626	28.2%
Mauritania	5,176	1,642	31.7%
Nigeria	391,372	61,029	15.6%
Pakistan*	399,530	98,080	24.5%
Sierra Leone	12,300	4,279	34.8%
Chad	36,118	7,021	19.4%
Total**	**1,125,296**	**233,347**	**20.7%**

*Indicates countries where female genital mutilation is not practiced and/or officially reported.

**Due to rounding, the totals presented in this table may differ slightly from the sum of individual country values.

We estimated that perinatal deaths attributable to the selected preconception risk factors generated productivity losses estimated at INT$21.3 billion in 2020 (Table 2). This foregone productivity results from the future loss of more than 7.9 million years of productive life. These productivity losses represent 0.7% of the fifteen countries’ combined 2020 GDP. Pakistan bears the highest economic burden (INT$11.1 billion, 0.9% of GDP), followed by Nigeria (INT$5.6 billion, 0.5% of GDP). These two countries account for about 78.6% of the total economic burden. Relative to its GDP, the productivity losses were the highest in Mali (1.5%) and Chad (1.0%). On average, the productivity losses represent a cost of $INT668 (SD = $INT432) per live birth.

**Table 2:** Present value in 2020 of foregone productivity due to perinatal mortality attributable to selected preconception risk factors (measured in $INT and as a percentage of 2020 GDP).

Country	Years of productive life lost (YPLLs)	Foregone productivity ($INT)	Foregone productivity (% of 2020 GDP)
Afghanistan*	467,200	602,016,365	0.7%
Burundi*	41,890	20,653,296	0.2%
Benin	86,184	178,383,446	0.4%
Côte d’Ivoire	373,618	1,197,157,129	0.8%
Guinea	200,829	337,105,709	0.9%
Gambia	36,774	49,324,612	0.9%
Kenya	284,161	772,850,997	0.3%
Liberia	65,478	56,649,791	0.8%
Lesotho*	8,754	11,488,527	0.2%
Mali	483,007	723,725,830	1.5%
Mauritania	41,829	141,833,900	0.6%
Nigeria	1,763,001	5,644,202,314	0.5%
Pakistan*	3,657,186	11,101,222,082	0.9%
Sierra Leone	111,560	109,159,881	0.8%
Chad	239,837	355,650,386	1.0%
Total**	**7,861,309**	**21,301,424,263**	**0.7%**

*Indicates countries where female genital mutilation is not practiced and/or officially reported.

**Due to rounding, the totals presented in this table may differ slightly from the sum of individual country values.

Table 3 shows the societal costs of perinatal mortality attributable to the preconception risk factors. Overall, we estimated these costs to be INT$51.0 billion in 2020. The estimate reflects the societal value of 10.8 million years of life lost (YLLs). As per the productivity losses, Pakistan and Nigeria generate most of the burden, with a contribution of 53.6% and 24.8%, respectively. On average, the societal losses represent a cost of $INT1,616 (SD = $INT1,101.9) per live birth.

**Table 3:** Present value in 2020 of societal costs due to perinatal mortality attributable to selected preconception risk factors (measured in $INT and as a percentage of 2020 GDP).

Country	Years of life lost (YLLs)	Societal costs ($INT)
Afghanistan*	622,242	1,725,471,607
Burundi*	56,049	42,283,869
Benin	116,877	394,677,229
Côte d’Ivoire	501,804	3,225,217,782
Guinea	262,692	863,203,531
Gambia	50,219	123,030,916
Kenya	390,386	1,973,094,734
Liberia	89,935	128,104,596
Lesotho*	9,537	51,238,952
Mali	649,012	1,443,629,656
Mauritania	60,621	341,567,181
Nigeria	2,395,999	12,660,517,988
Pakistan*	5,107,449	27,364,841,228
Sierra Leone	148,173	226,673,596
Chad	308,816	464,341,633
Total**	**10,769,812**	**51,027,894,497**

*Indicates countries where female genital mutilation is not practiced and/or officially reported.

**Due to rounding, the totals presented in this table may differ slightly from the sum of individual country values.

On average, adolescent pregnancy made up 29.0% of the economic burden (Table 4). It was the largest contributor, followed by short birth interval (26.1%) and pre-pregnancy overweight and obesity (24.6%). It is important to note that the average contribution of short birth intervals was strongly driven by the distribution in Pakistan, where it accounts for 41.7% of the economic burden. 6.0% of the foregone productivity or societal losses were generated by intimate partner violence before pregnancy. The average contribution of female genital mutilation was 14.3%, with substantial variations between countries.

**Table 4:** Distribution of the economic burden by preconception risk factor.

Country	Adolescent pregnancy	Short birth interval	Intimate partner violence	Pre-pregnancy overweight and obesity	Female genital mutilation type II and III
Afghanistan*	31.6%	25.9%	9.5%	32.9%	0.0%
Burundi*	37.9%	20.4%	22.2%	19.5%	0.0%
Benin	38.8%	11.1%	8.4%	27.1%	14.6%
Côte d’Ivoire	32.6%	7.5%	4.9%	18.0%	37.1%
Guinea	20.7%	4.6%	5.4%	11.6%	57.7%
Gambia	15.5%	4.2%	3.0%	16.6%	60.7%
Kenya	25.8%	9.4%	6.6%	36.9%	21.2%
Liberia	36.2%	6.9%	9.6%	20.3%	27.0%
Lesotho*	9.3%	8.9%	11.7%	70.1%	0.0%
Mali	24.9%	9.7%	6.1%	12.0%	47.3%
Mauritania	19.1%	13.1%	2.1%	26.3%	39.5%
Nigeria	35.7%	16.9%	7.7%	22.6%	17.0%
Pakistan*	25.7%	41.7%	4.1%	28.6%	0.0%
Sierra Leone	16.8%	3.6%	6.1%	10.4%	63.1%
Chad	30.9%	18.8%	7.9%	7.4%	35.1%
**Total**	**29.0%**	**26.1%**	**6.0%**	**24.6%**	**14.3%**

*Indicates countries where female genital mutilation is not practiced and/or officially reported.

Table 5 shows the results of our sensitivity analysis. We re-conducted the analysis using the lower and upper bounds of the relative risks. The total foregone productivity varied from INT$11.2 (−47.4%) billion to INT$37.1 billion (+74.2%), and the societal costs from INT$27.0 billion (−47.1%) to INT$88.3 billion (+73.1%). Using 5% and 7% discount rates reduced the productivity losses by 46.5% and 69.5%, respectively. The societal losses were reduced by 33.1% with a 5% discount rate, and 50.8% with a 7% discount rate. These results indicate that the magnitude of the economic burden is dependent on the relative risks and the discount rates utilised.

**Table 5:** Sensitivity analysis.

Country	Present value in 2020 of foregone productivity ($INT, Billion)	Present value in 2020 of societal costs ($INT, Billion)
**Base Scenario (BS)**	**21.3**	**51.0**
**Mean (BS)**		
Lower Bound	11.2	27.0
Upper Bound	37.1	88.3
**Discount Rate: 3% (BS)**		
Discount Rate: 5%	11.4	34.1
Discount Rate: 7%	6.5	25.1

## Discussion

The study results indicated that more than 233,000 cases of perinatal deaths were attributable to the five preconception risk factors in 2020, equivalent to 20.7% of all cases reported in the fifteen countries. This mortality burden, which translates into 10.8 million years of life lost and 7.9 million years of productive life lost, had profound economic and societal implications. In 2020, the foregone productivity was estimated at $INT21.3 billion, representing 0.7% of the fifteen countries’ combined GDP. The use of a value of a statistical life-year approach enabled us to capture the economic burden from a societal perspective, revealing a cost of $INT51.0 billion.

To our knowledge, this study is the first to assess the economic burden of various preconception risk factors through their impact on perinatal mortality in LMICs. Therefore, we do not have prior studies to compare our findings directly. To estimate the PAF of early neonatal deaths for adolescent pregnancy and short birth interval, we used preterm births, preterm births with small-for-gestational-age, and small-for-gestational-age births as intermediate outcomes. These outcomes are among the main causes of early neonatal mortality with congenital anomalies, birth asphyxia, and infections, such as pneumonia or neonatal sepsis [36]. We found that 9.9% of preterm births were attributable to adolescent pregnancy, and 3.9% to short birth spacing in the thirteen Sub-Saharan African countries included in our model. Additionally, 5.9% of small-for-gestational-age births were attributable to adolescent pregnancy and 2.4% to short birth spacing. These estimates are higher than the PAFs reported in two recently published studies [26,37]. These differences might be partly because, unlike our study, these two studies did not consider preterm births with small-for-gestational-age as an independent category. Additionally, those studies adjusted for a larger number of potential confounders, which could further explain the lower PAFs they reported. Our estimates of the PAF of stillbirths attributable to pre-pregnancy overweight and obesity are coherent with those of a previous study conducted in Australia [38]. Nevertheless, this study used maternal body mass index and not pre-pregnancy body mass index, which, once again, limits the comparison. With regard to the economic burden analysis, we did not find studies with which a comparison would have been relevant. However, it is worth mentioning the study by Kirigia et al. [12], in which the authors estimated the non-health GDP loss due to under-five mortality in 47 African countries to be INT$150.3 billion in 2013. Similar to our study, a large share of the economic burden was borne by Nigeria.

We found that PAFs varied substantially between countries, with the lowest being found in Burundi (7.5%) and the highest in Sierra Leone (34.8%). As mentioned above, the variations are amplified by the fact that the impact of female genital mutilation was null in four out of the fifteen selected countries. Female genital mutilation is reportedly not practised in Burundi and Lesotho [39]. In Pakistan, the practice is not officially reported, though it is believed that it exists or has existed marginally, likely confined to a few isolated communities [40]. The situation in Afghanistan remains unclear. In 2015, a study conducted in Iran found that women of Afghan origin had higher risks of experiencing female genital mutilation [41], but no study has comprehensively assessed the prevalence of this practice in Afghanistan, which is not officially reported. The observed variations in PAFs also reflect substantial differences in the magnitude of exposure to the selected preconception risk factors. These disparities are influenced by a complex interplay of socio-economic, cultural, and healthcare-related factors that vary across different regions and populations. Since PAFs are highly sensitive to risk exposure levels, even minor differences in prevalence rates can lead to significant differences between countries, subsequently influencing the economic burden.

In this study, we used preterm birth with or without small-for-gestational-age as intermediate outcomes to capture the influence of early and repeated pregnancies and intimate partner violence before pregnancy on perinatal mortality. Research has shown that babies born prematurely and/or small for gestational age are more likely to die within the first seven days of life due to higher susceptibility to complications [42]. Several explanations have been advanced to explain why children born from adolescent mothers are at higher risks of prematurity and small for gestational age, including increased competition for nutrients between the mother and the foetus, inadequate maternal nutritional intake, physiological immaturity, higher prevalence of anaemia, and increased likelihood of pregnancy-induced hypertension and other obstetric complications in adolescents [43]. Short birth intervals have also been associated with increased risks of preterm births and small-for-gestational-age births [44]. Pregnancies that are closely spaced together impose physiological stress on mothers that hinders adequate recovery and replenishment of nutritional reserves, impairing foetal growth and maturation. In women with previous caesarean deliveries, short birth intervals may lead to incomplete healing of the uterine scar, resulting in complications such as preterm labour. Additionally, shorter interpregnancy intervals may elevate the probability of maternal infections being transmitted to the foetus due to the maternal immune system having insufficient time to fully recover between pregnancies. The mechanisms linking abuse before pregnancy and preterm labour remain unclear. One possible explanation is that women with such a history are more likely to engage in behaviours associated with increased risks of prematurity, including smoking, drug abuse, or alcohol use, and to receive inadequate prenatal care [22,45]. We also hypothesise that exposure to abuse is more likely to continue during pregnancy if initiated beforehand, further compounding the risk of prematurity and adverse pregnancy outcomes.

Female genital mutilation persists in many countries and is driven by a complex interplay of cultural traditions, sociocultural norms, and economic pressures. The mechanisms by which female genital mutilation might cause adverse obstetric outcomes, including stillbirth, are not fully understood. It is possible that the presence of scar tissue from female genital mutilation increases the risks of obstruction and tears during labour, which can prolong the second stage of labour. This, in turn, increases the risks of perineal injury and postpartum haemorrhage, ultimately leading to higher risks of stillbirth [18]. Pre-pregnancy overweight and obesity have also been linked to higher risks of stillbirths. The biological pathways include increased risks of diabetes, hypertension, and hyperlipidaemia, as well as more frequent apnoeic hypoxemia events that reduce blood flow to the foetus. Overweight and obese women may also have a reduced perception of foetal movement, which delays timely medical intervention and increases the risk of adverse outcomes [21].

Our findings suggest that investing in interventions and policies that address the selected risk factors may be an avenue to reduce perinatal mortality. However, it is important to recall that the PAFs reported in our study represent the proportion of perinatal deaths that could be prevented if the associated risk factors were entirely eliminated, which is an idealised scenario. In reality, completely eradicating these risk factors is highly unlikely due to a variety of challenges, including socioeconomic constraints, health system limitations, cultural practices, and individual behaviours. Therefore, while our findings underscore the potential impact of targeting these five preconception risk factors, they should be interpreted cautiously. Achieving significant reductions in perinatal mortality through the preconception pathway will require multifaceted approaches that combine public health policies, education, healthcare improvements, and social support systems.

This study has several limitations that must be acknowledged. First, we focused on five preconception risk factors, although many others, such as malnutrition, infections, poor mental health, or substance abuse, are also linked to perinatal mortality. Similarly, we did not account for other important outcomes, including maternal mortality, pregnancy and childbirth-related complications or long-term impacts like childhood stunting, which also contribute to economic and societal losses. We chose to limit this analysis to perinatal mortality to maintain a clear narrative but also because it is likely to be the main contributor to the economic burden of poor preconception health. Second, the analysis was limited to fifteen countries with available and relatively recent DHS, restricting the generalizability of our findings to other LMICs with different socio-economic contexts. Third, we did not adjust the PAFs for confounding risk factors. However, we minimised this limitation by using adjusted relative risks wherever possible. Fourth, our model assumed that all women have an equal chance of becoming pregnant, regardless of their exposure to overweight and obesity, female genital mutilation, and intimate partner violence. This assumption overlooks the nuanced reality that some risk factors may influence fertility rates, either enhancing or diminishing a woman’s likelihood of conception. Unfortunately, it was not possible to differentiate fertility rates in exposed and not-exposed women due to data limitations. Fifth, we did not factor in potential changes in the GDP per working-age population or the VSLY over time. Introducing such projections could have increased uncertainty around our estimates, so we adopted a more conservative approach by not adjusting these parameters over time.

Despite these limitations, and given the scarce published evidence in this area, our study provides new insights into the economics of preconception health and care in LMICs. It might be beneficial to investigate this further by extending the scope of this study, whether in terms of risk factors, outcomes or selected countries. In addition, further research should focus on building an investment case to assess the economic value of investing in preconception health and care, which could guide policymaking and resource allocation in LMICs.

In LMICs, a substantial proportion of perinatal deaths is attributable to risk factors that can be addressed before pregnancy starts, such as early and rapidly repeated pregnancies, overweight and obesity or exposure to violence. In turn, these perinatal deaths cause significant economic losses at the country level. Results from this study provide insights into the profound and long-term economic implications that arise from inaction on preconception health and care. They also highlight the potential of preconception care interventions and policies for curbing the burden of stillbirths and neonatal mortality while contributing to the achievement of the SDGs. To stimulate interest from policymakers and the public, it is essential to pursue research efforts on the cost and benefits of preconception care in LMICs.

## Supporting information

S1 Appendix Supplementary information including methods, data inputs, and model assumptions. (DOCX)
