# Supplementary material for: The economic burden of perinatal mortality due to inaction on preconception health in low and middle-income countries: A population attributable fraction and economic impact analysis
Source: PLoS One. 2025 Jul 3;20(7):e0325086. doi: 10.1371/journal.pone.0325086 (PMC12225852; doi:10.1371/journal.pone.0325086)
Supplement: S1 Appendix — (DOCX) [file pone.0325086.s001.docx]

**S1 Appendix. Supplementary information including methods, data inputs, and model assumptions**

**The economic burden of perinatal mortality due to inaction on preconception health in low and middle-income countries: a population attributable fraction and economic impact analysis**

Sébastien Poix, Patrick O’Donnell, Khalifa Elmusharaf

**Table of contents**

[A. Guidelines for Accurate and Transparent Health Estimates Reporting (GATHER) checklist 3](#_Toc201456007)

[B. Prevalence rates of the selected preconception risk factors 6](#_Toc201456008)

[C. Summary of the relative risks used in the model 10](#_Toc201456009)

[D. Description of the methodology used to convert ORs to RRs 12](#_Toc201456010)

[E. Description of the methodology used to estimate the PAF of perinatal mortality for the selected preconception risk factors 14](#_Toc201456011)

[F. Main parameters used to estimate the economic burden associated with perinatal mortality 16](#_Toc201456012)

[References 18](#_Toc201456013)

1. Guidelines for Accurate and Transparent Health Estimates Reporting (GATHER) checklist

| **#** | **Checklist item** |  |
| --- | --- | --- |
| **Objectives and funding** | | |
| 1 | Define the indicator(s), populations (including age, sex, and geographic entities), and time period(s) for which estimates were made. | The indicator(s), populations and time periods for which estimates were made are described in the sections “Approach”, “Selected countries”, “Population attributable fraction analysis”, and “Economic impact analysis”. |
| 2 | List the funding sources for the work. | As stated in “Funding”, this research was conducted without any specific grant from funding agencies in the public, commercial, or not-for-profit sectors. |
| **Data inputs** | | |
| *For all data inputs from multiple sources that are synthesised as part of the study:* | | |
| 3 | Describe how the data were identified and how the data were accessed. | We used secondary data from online database, health surveys and the literature. The data sources are provided throughout the “Methods” section and in the supplementary materials. |
| 4 | Specify the inclusion and exclusion criteria· Identify all ad-hoc exclusions. | The inclusion and exclusion criteria used to select the countries are provided in the section “Selected countries”. |
| 5 | Provide information about all included data sources and their main characteristics· For each data source used, report reference information or contact name/institution, population represented, data collection method, year(s) of data collection, sex and age range, diagnostic criteria or measurement method, and sample size, as relevant. | The data sources are provided throughout the “Methods” section and in the supplementary materials. |
| 6 | Identify and describe any categories of input data that have potentially important biases (eg, based on characteristics listed in item 5). | In the “Discussion”, we identified potential biases related to prevalence data and relative risks used in the model. |
| *For data inputs that contribute to the analysis but were not synthesised as part of the study:* | | |
| 7 | Describe and give sources for any other data inputs. | All data sources are provided throughout the “Methods” section and/or in the supplementary materials. |
| *For all data inputs:* | | |
| 8 | Provide all data inputs in a file format from which data can be efficiently extracted (eg, a spreadsheet rather than a PDF), including all relevant meta-data listed in item 5. For any data inputs that cannot be shared because of ethical or legal reasons, such as third-party ownership, provide a contact name or the name of the institution that retains the right to the data. | The main data inputs are provided in the supplementary information. The data nor listed in the supplementary materials (number of births stratified by 5-year age group, neonatal mortality rate, stillbirths rate, and percent of neonatal deaths occurring within seven days after birth) are publicly accessible and the sources are given in throughout the “Methods” section. |
| 9 | Provide a conceptual overview of the data analysis method· A diagram may be helpful. | The conceptual framework is presented as a diagram in Figure 1. |
| 10 | Provide a detailed description of all steps of the analysis, including mathematical formulae. This description should cover, as relevant, data cleaning, data pre-processing, data adjustments and weighting of data sources, and mathematical or statistical model(s). | All steps of the analysis are presented in the section “population attributable fraction analysis” and “economic impact analysis”. The detailed processus used to adjust the PAFs and convert ORs into RRs are presented in the supplementary information. |
| 11 | Describe how candidate models were evaluated and how the final model(s) were selected. | This study did not involve the evaluation of multiple candidate models and the selection of a final model. We conducted the analysis using a unique model, which was deemed appropriate for the study objectives and data available. |
| 12 | Provide the results of an evaluation of model performance, if done, as well as the results of any relevant sensitivity analysis. | We did not assess the performance of the model as part of this study· However, we conducted a sensitivity analysis whose results are reported in the section “Sensitivity analysis”. |
| 13 | Describe methods of calculating uncertainty of the estimates. State which sources of uncertainty were, and were not, accounted for in the uncertainty analysis. | To assess uncertainty of the results, we calculated the PAFs using the lower and upper bounds of the relative risks obtained from the literature. The results are presented in the section “Sensitivity analysis”. |
| 14 | State how analytical or statistical source code used to generate estimates can be accessed. | The model was created in an Excel spreadsheet and is available upon request, as stated in “Data availability”. |
| **Results and discussion** | | |
| 15 | Provide published estimates in a file format from which data can be efficiently extracted. | Data are reported in Table 1, 2, 3, 4, and 5. All estimates can be efficiently and easily from these tables. |
| 16 | Report a quantitative measure of the uncertainty of the estimates (eg, uncertainty intervals). | For clarity, we reported estimates without uncertainty intervals. However, the use of lower and upper bounds of the relative risks, as part of the sensitivity analysis, provides a measure of uncertainty. |
| 17 | Interpret results in light of existing evidence. If updating a previous set of estimates, describe the reasons for changes in estimates. | In the “Discussion” section, we interpreted our findings in light of existing evidence. |
| 18 | Discuss limitations of the estimates. Include a discussion of any modelling assumptions or data limitations that affect interpretation of the estimates. | We discuss the main limitations of our studies in the “Discussion” section. |

1. Prevalence rates of the selected preconception risk factors

**Note:** Prevalence rates are represented in the table as decimal values (e.g. 0·77 represents 77·0%)

| **First births (%)** |  | **AFG** | **BDI** | **BEN** | **CIV** | **GIN** | **GMB** | **KEN** | **LIB** | **LSO** | **MLI** | **MRT** | **NIG** | **PAK** | **SLE** | **TCD** |
| --- | --- | --- | --- | --- | --- | --- | --- | --- | --- | --- | --- | --- | --- | --- | --- | --- |
|  | 15-19 | 0·77 | 0·91 | 0·84 | 0·84 | 0·77 | 0·87 | 0·87 | 0·85 | 0·93 | 0·75 | 0·72 | 0·78 | 0·82 | 0·87 | 0·71 |
|  | 20-24 | 0·35 | 0·47 | 0·44 | 0·45 | 0·28 | 0·44 | 0·56 | 0·38 | 0·63 | 0·29 | 0·35 | 0·35 | 0·44 | 0·43 | 0·20 |
|  | 25-29 | 0·09 | 0·10 | 0·11 | 0·17 | 0·09 | 0·15 | 0·20 | 0·19 | 0·24 | 0·06 | 0·16 | 0·12 | 0·21 | 0·13 | 0·04 |
|  | 30-34 | 0·02 | 0·04 | 0·03 | 0·06 | 0·03 | 0·05 | 0·05 | 0·02 | 0·14 | 0·02 | 0·06 | 0·07 | 0·08 | 0·02 | 0·00 |
|  | 35-39 | 0·02 | 0·01 | 0·01 | 0·02 | 0·01 | 0·01 | 0·01 | 0·00 | 0·01 | 0·00 | 0·02 | 0·02 | 0·02 | 0·01 | 0·00 |
|  | 40-44 | 0·00 | 0·01 | 0·00 | 0·03 | 0·00 | 0·00 | 0·01 | 0·00 | 0·02 | 0·00 | 0·01 | 0·01 | 0·02 | 0·00 | 0·00 |
|  | 45-49 | 0·00 | 0·00 | 0·00 | 0·06 | 0·01 | 0·02 | 0·00 | 0·00 | 0·00 | 0·02 | 0·04 | 0·00 | 0·00 | 0·00 | 0·03 |
| **Non-first births (%)** |  | **AFG** | **BDI** | **BEN** | **CIV** | **GIN** | **GMB** | **KEN** | **LIB** | **LSO** | **MLI** | **MRT** | **NIG** | **PAK** | **SLE** | **TCD** |
|  | 15-19 | 0·23 | 0·09 | 0·16 | 0·16 | 0·23 | 0·13 | 0·13 | 0·15 | 0·07 | 0·25 | 0·28 | 0·22 | 0·18 | 0·13 | 0·29 |
|  | 20-24 | 0·65 | 0·54 | 0·56 | 0·55 | 0·72 | 0·56 | 0·44 | 0·62 | 0·37 | 0·71 | 0·65 | 0·65 | 0·57 | 0·58 | 0·80 |
|  | 25-29 | 0·91 | 0·91 | 0·89 | 0·83 | 0·91 | 0·86 | 0·80 | 0·81 | 0·76 | 0·94 | 0·84 | 0·88 | 0·80 | 0·87 | 0·96 |
|  | 30-34 | 0·98 | 0·96 | 0·97 | 0·94 | 0·97 | 0·95 | 0·95 | 0·98 | 0·86 | 0·98 | 0·94 | 0·93 | 0·92 | 0·98 | 1·00 |
|  | 35-39 | 0·98 | 0·99 | 0·99 | 0·98 | 0·99 | 0·99 | 0·99 | 1·00 | 1·00 | 1·00 | 0·98 | 0·98 | 0·98 | 0·99 | 1·00 |
|  | 40-44 | 1·00 | 0·99 | 1·00 | 0·97 | 1·00 | 1·00 | 0·99 | 1·00 | 0·98 | 1·00 | 0·99 | 0·99 | 0·98 | 1·00 | 1·00 |
|  | 45-49 | 1·00 | 1·00 | 1·00 | 0·94 | 0·99 | 0·98 | 1·00 | 1·00 | 1·00 | 0·98 | 0·96 | 1·00 | 1·00 | 1·00 | 0·97 |
| **Births in women < 19 years old (%)** |  | **AFG** | **BDI** | **BEN** | **CIV** | **GIN** | **GMB** | **KEN** | **LIB** | **LSO** | **MLI** | **MRT** | **NIG** | **PAK** | **SLE** | **TCD** |
|  | 15-19 | 0·13 | 0·08 | 0·13 | 0·17 | 0·18 | 0·10 | 0·13 | 0·22 | 0·02 | 0·20 | 0·13 | 0·14 | 0·08 | 0·18 | 0·17 |
| **Births whose previous birth interval is 7-17 months (%)** |  | **AFG** | **BDI** | **BEN** | **CIV** | **GIN** | **GMB** | **KEN** | **LIB** | **LSO** | **MLI** | **MRT** | **NIG** | **PAK** | **SLE** | **TCD** |
|  | 15-19 | 0·37 | 0·00 | 0·14 | 0·06 | 0·06 | 0·16 | 0·19 | 0·05 | 0·00 | 0·18 | 0·12 | 0·12 | 0·33 | 0·15 | 0·16 |
|  | 20-24 | 0·18 | 0·10 | 0·07 | 0·08 | 0·05 | 0·06 | 0·12 | 0·07 | 0·06 | 0·12 | 0·14 | 0·11 | 0·32 | 0·06 | 0·12 |
|  | 25-29 | 0·12 | 0·06 | 0·05 | 0·06 | 0·04 | 0·04 | 0·07 | 0·06 | 0·05 | 0·08 | 0·12 | 0·09 | 0·19 | 0·05 | 0·11 |
|  | 30-34 | 0·10 | 0·06 | 0·04 | 0·04 | 0·04 | 0·03 | 0·06 | 0·05 | 0·03 | 0·07 | 0·09 | 0·08 | 0·17 | 0·05 | 0·10 |
|  | 35-39 | 0·08 | 0·05 | 0·04 | 0·05 | 0·05 | 0·03 | 0·05 | 0·04 | 0·01 | 0·06 | 0·09 | 0·07 | 0·11 | 0·03 | 0·11 |
|  | 40-44 | 0·06 | 0·05 | 0·03 | 0·04 | 0·05 | 0·01 | 0·04 | 0·04 | 0·02 | 0·06 | 0·07 | 0·06 | 0·13 | 0·05 | 0·13 |
|  | 45-49 | 0·06 | 0·03 | 0·04 | 0·06 | 0·06 | 0·01 | 0·05 | 0·12 | 0·00 | 0·07 | 0·08 | 0·06 | 0·11 | 0·06 | 0·08 |
| **Woman with a BMI between 25·0 and 29·9 (%)** |  | **AFG** | **BDI** | **BEN** | **CIV** | **GIN** | **GMB** | **KEN** | **LIB** | **LSO** | **MLI** | **MRT** | **NIG** | **PAK** | **SLE** | **TCD** |
|  | 15-19 | 0·24 | 0·04 | 0·06 | 0·12 | 0·09 | 0·10 | 0·10 | 0·09 | 0·15 | 0·10 | 0·20 | 0·07 | 0·21 | 0·10 | 0·04 |
|  | 20-24 | 0·24 | 0·06 | 0·11 | 0·18 | 0·17 | 0·15 | 0·19 | 0·19 | 0·22 | 0·13 | 0·26 | 0·13 | 0·22 | 0·20 | 0·06 |
|  | 25-29 | 0·24 | 0·07 | 0·19 | 0·24 | 0·18 | 0·24 | 0·28 | 0·26 | 0·30 | 0·20 | 0·32 | 0·20 | 0·33 | 0·22 | 0·08 |
|  | 30-34 | 0·24 | 0·08 | 0·21 | 0·29 | 0·25 | 0·31 | 0·29 | 0·32 | 0·27 | 0·24 | 0·32 | 0·22 | 0·29 | 0·23 | 0·12 |
|  | 35-39 | 0·24 | 0·06 | 0·24 | 0·26 | 0·26 | 0·32 | 0·32 | 0·40 | 0·30 | 0·23 | 0·29 | 0·26 | 0·31 | 0·25 | 0·14 |
|  | 40-44 | 0·24 | 0·07 | 0·23 | 0·26 | 0·20 | 0·34 | 0·28 | 0·30 | 0·32 | 0·30 | 0·28 | 0·23 | 0·36 | 0·21 | 0·15 |
|  | 45-49 | 0·31 | 0·06 | 0·25 | 0·26 | 0·25 | 0·30 | 0·32 | 0·31 | 0·30 | 0·24 | 0·30 | 0·24 | 0·35 | 0·25 | 0·12 |
| **Woman with a BMI ≥ 30 (%)** |  | **AFG** | **BDI** | **BEN** | **CIV** | **GIN** | **GMB** | **KEN** | **LIB** | **LSO** | **MLI** | **MRT** | **NIG** | **PAK** | **SLE** | **TCD** |
|  | 15-19 | 0·15 | 0·00 | 0·01 | 0·04 | 0·02 | 0·03 | 0·02 | 0·01 | 0·03 | 0·03 | 0·09 | 0·01 | 0·04 | 0·02 | 0·01 |
|  | 20-24 | 0·15 | 0·00 | 0·03 | 0·04 | 0·04 | 0·09 | 0·06 | 0·05 | 0·11 | 0·05 | 0·17 | 0·03 | 0·09 | 0·04 | 0·01 |
|  | 25-29 | 0·15 | 0·02 | 0·07 | 0·12 | 0·09 | 0·12 | 0·12 | 0·15 | 0·22 | 0·07 | 0·29 | 0·08 | 0·18 | 0·08 | 0·02 |
|  | 30-34 | 0·31 | 0·03 | 0·14 | 0·17 | 0·12 | 0·20 | 0·21 | 0·19 | 0·26 | 0·11 | 0·33 | 0·13 | 0·24 | 0·10 | 0·03 |
|  | 35-39 | 0·31 | 0·04 | 0·19 | 0·23 | 0·13 | 0·21 | 0·24 | 0·18 | 0·32 | 0·14 | 0·44 | 0·16 | 0·28 | 0·12 | 0·05 |
|  | 40-44 | 0·31 | 0·02 | 0·18 | 0·20 | 0·15 | 0·27 | 0·24 | 0·20 | 0·34 | 0·16 | 0·45 | 0·17 | 0·27 | 0·18 | 0·04 |
|  | 45-49 | 0·29 | 0·03 | 0·19 | 0·22 | 0·14 | 0·31 | 0·25 | 0·24 | 0·37 | 0·14 | 0·43 | 0·21 | 0·31 | 0·13 | 0·06 |
| **Ever-married women who have experienced physical or sexual violence committed by their husband/partner in the last 12 months (%)** |  | **AFG** | **BDI** | **BEN** | **CIV** | **GIN** | **GMB** | **KEN** | **LIB** | **LSO** | **MLI** | **MRT** | **NIG** | **PAK** | **SLE** | **TCD** |
|  | 15-19 | 0·29 | 0·38 | 0·14 | 0·17 | 0·17 | 0·14 | 0·14 | 0·58 | 0·20 | 0·21 | 0·08 | 0·13 | 0·17 | 0·43 | 0·15 |
|  | 20-24 | 0·41 | 0·34 | 0·13 | 0·16 | 0·21 | 0·16 | 0·19 | 0·53 | 0·19 | 0·23 | 0·08 | 0·19 | 0·11 | 0·49 | 0·19 |
|  | 25-29 | 0·49 | 0·29 | 0·16 | 0·17 | 0·24 | 0·13 | 0·21 | 0·38 | 0·18 | 0·24 | 0·06 | 0·16 | 0·14 | 0·49 | 0·19 |
|  | 30-34 | 0·49 | 0·29 | 0·16 | 0·17 | 0·23 | 0·10 | 0·20 | 0·33 | 0·16 | 0·23 | 0·08 | 0·14 | 0·20 | 0·42 | 0·20 |
|  | 35-39 | 0·49 | 0·27 | 0·13 | 0·10 | 0·22 | 0·09 | 0·21 | 0·27 | 0·15 | 0·19 | 0·05 | 0·13 | 0·16 | 0·37 | 0·19 |
|  | 40-44 | 0·45 | 0·23 | 0·12 | 0·09 | 0·19 | 0·06 | 0·17 | 0·33 | 0·13 | 0·15 | 0·03 | 0·11 | 0·12 | 0·33 | 0·13 |
|  | 45-49 | 0·52 | 0·22 | 0·11 | 0·08 | 0·16 | 0·05 | 0·16 | 0·18 | 0·11 | 0·14 | 0·08 | 0·07 | 0·11 | 0·22 | 0·14 |
| **Women who have experienced female genital mutilation (%)** |  | **AFG** | **BDI** | **BEN** | **CIV** | **GIN** | **GMB** | **KEN** | **LIB** | **LSO** | **MLI** | **MRT** | **NIG** | **PAK** | **SLE** | **TCD** |
|  | 15-19 | NA | NA | 0·02 | 0·31 | 0·92 | 0·73 | 0·09 | 0·20 | NA | 0·86 | 0·56 | 0·14 | NA | 0·61 | 0·32 |
|  | 20-24 | NA | NA | 0·04 | 0·35 | 0·94 | 0·72 | 0·10 | 0·21 | NA | 0·87 | 0·61 | 0·16 | NA | 0·81 | 0·38 |
|  | 25-29 | NA | NA | 0·07 | 0·37 | 0·95 | 0·75 | 0·13 | 0·26 | NA | 0·91 | 0·68 | 0·18 | NA | 0·88 | 0·42 |
|  | 30-34 | NA | NA | 0·10 | 0·40 | 0·95 | 0·71 | 0·16 | 0·37 | NA | 0·90 | 0·67 | 0·20 | NA | 0·91 | 0·41 |
|  | 35-39 | NA | NA | 0·10 | 0·45 | 0·96 | 0·72 | 0·19 | 0·44 | NA | 0·88 | 0·67 | 0·22 | NA | 0·93 | 0·40 |
|  | 40-44 | NA | NA | 0·12 | 0·45 | 0·97 | 0·72 | 0·24 | 0·47 | NA | 0·91 | 0·68 | 0·27 | NA | 0·94 | 0·42 |
|  | 45-49 | NA | NA | 0·12 | 0·47 | 0·98 | 0·74 | 0·23 | 0·54 | NA | 0·89 | 0·74 | 0·31 | NA | 0·95 | 0·39 |
| **Women who have experienced female genital mutilation type II as a proportion of all women who have experienced female genital mutilation (%)** |  | **AFG** | **BDI** | **BEN** | **CIV** | **GIN** | **GMB** | **KEN** | **LIB** | **LSO** | **MLI** | **MRT** | **NIG** | **PAK** | **SLE** | **TCD** |
|  | 15-19 | NA | NA | 0·61 | 0·68 | 0·56 | 0·74 | 0·67 | 0·57 | NA | 0·38 | 0·51 | 0·31 | NA | 0·83 | 0·39 |
|  | 20-24 | NA | NA | 0·70 | 0·69 | 0·59 | 0·71 | 0·63 | 0·59 | NA | 0·40 | 0·53 | 0·36 | NA | 0·83 | 0·44 |
|  | 25-29 | NA | NA | 0·66 | 0·71 | 0·58 | 0·73 | 0·70 | 0·60 | NA | 0·39 | 0·57 | 0·41 | NA | 0·85 | 0·39 |
|  | 30-34 | NA | NA | 0·70 | 0·70 | 0·61 | 0·71 | 0·70 | 0·61 | NA | 0·43 | 0·60 | 0·41 | NA | 0·83 | 0·45 |
|  | 35-39 | NA | NA | 0·71 | 0·70 | 0·57 | 0·74 | 0·70 | 0·62 | NA | 0·43 | 0·57 | 0·47 | NA | 0·86 | 0·43 |
|  | 40-44 | NA | NA | 0·69 | 0·73 | 0·58 | 0·79 | 0·73 | 0·63 | NA | 0·43 | 0·58 | 0·43 | NA | 0·85 | 0·51 |
|  | 45-49 | NA | NA | 0·70 | 0·80 | 0·56 | 0·75 | 0·77 | 0·63 | NA | 0·45 | 0·52 | 0·48 | NA | 0·85 | 0·43 |
| **Women who have experienced female genital mutilation type III as a proportion of all women who have experienced female genital mutilation (%)** |  | **AFG** | **BDI** | **BEN** | **CIV** | **GIN** | **GMB** | **KEN** | **LIB** | **LSO** | **MLI** | **MRT** | **NIG** | **PAK** | **SLE** | **TCD** |
|  | 15-19 | NA | NA | 0·09 | 0·10 | 0·08 | 0·12 | 0·13 | 0·09 | NA | 0·06 | 0·07 | 0·03 | NA | 0·13 | 0·12 |
|  | 20-24 | NA | NA | 0·12 | 0·09 | 0·09 | 0·15 | 0·13 | 0·10 | NA | 0·08 | 0·10 | 0·04 | NA | 0·13 | 0·10 |
|  | 25-29 | NA | NA | 0·15 | 0·09 | 0·10 | 0·19 | 0·12 | 0·11 | NA | 0·09 | 0·10 | 0·05 | NA | 0·11 | 0·09 |
|  | 30-34 | NA | NA | 0·11 | 0·09 | 0·10 | 0·21 | 0·13 | 0·11 | NA | 0·09 | 0·10 | 0·04 | NA | 0·13 | 0·10 |
|  | 35-39 | NA | NA | 0·12 | 0·07 | 0·09 | 0·18 | 0·12 | 0·11 | NA | 0·09 | 0·12 | 0·08 | NA | 0·11 | 0·08 |
|  | 40-44 | NA | NA | 0·12 | 0·07 | 0·12 | 0·15 | 0·11 | 0·10 | NA | 0·08 | 0·09 | 0·06 | NA | 0·12 | 0·09 |
|  | 45-49 | NA | NA | 0·16 | 0·08 | 0·13 | 0·18 | 0·08 | 0·11 | NA | 0·11 | 0·12 | 0·09 | NA | 0·11 | 0·07 |
| AFG: Afghanistan, BDI: Burundi, BEN: Benin, CIV: Côte d’Ivoire, GIN: Guinea, KEN: Kenya, LBR: Liberia, LSO: Lesotho, MLI: Mali, MRT: Mauritania, NIG: Nigeria, PAK: Pakistan, SLE: Sierra Leone, TCD: Tchad  NA: Not applicable | | | | | | | | | | | | | | | | |

1. Summary of the relative risks used in the model

| **Study** | **Risk factor** | **Outcomes** | **Relative risks (95% CI)** | **Reference** | **Number of studies** |
| --- | --- | --- | --- | --- | --- |
| Kozuki and colleagues^1^ | Nulliparous / Age > 18 | Term-SGA | 1·52* | 18-34 years old | 14 |
|  | Nulliparous / Age > 18 | Preterm-AGA | 1·75* |  | 13 |
|  | Nulliparous / Age > 18 | Preterm-SGA | 3·14* |  | 14 |
| Kozuki and colleagues^2^ | Birth interval > 18 months | Term-SGA | 1·41* | Birth interval ≥ 24 months | 5 |
|  | Birth interval > 18 months | Preterm-AGA | 1·49* |  | 5 |
|  | Birth interval > 18 months | Preterm-SGA | 3·03* |  | 5 |
| Nesari and colleagues^3^ | Maternal abuse (< 12 months before pregnancy) | Preterm | 1·25 (1·08-1·49)^§ ‡^ | No abuse | 4 |
| Katz and colleagues^4^ | Term-SGA (Africa) | Early neonatal mortality | 2·16 (1·61-2·90)^†^ | Appropriate for gestational age (AGA) | 8 |
|  | Preterm-AGA (Africa) |  | 7·71 (3·15-18·86)^†^ |  | 8 |
|  | Preterm-SGA (Africa) |  | 8·36 (5·36-13·04)^†^ |  | 8 |
|  | Term-SGA (Asia) | Early neonatal mortality | 3·33 (2·37-4·66)^†^ |  | 8 |
|  | Preterm-AGA (Asia) |  | 6·17 (3·82-9·99)^†^ |  | 8 |
|  | Preterm-SGA (Asia) |  | 14·66 (10·68-20·13)^†^ |  | 8 |
| Banks and colleagues^5^ | Female genital mutilation type II | Inpatient perinatal death | 1·32 (1·08-1·62) | No FGM | -^‡^ |
|  | Female genital mutilation type III |  | 1·55 (1·12-2·16) |  | -^‡^ |
| Vats and colleagues^6^ | Pre-pregnancy overweight | Stillbirth | 1·23 (1·12-1·36)^§^ | Normal weight | 15 |
|  | Pre-pregnancy obesity |  | 1·54 (1·35-1·74)^§^ |  | 18 |
| *The adjusted relative risks relative risks were not published, but were calculated for use in the Lives Saved Tool (LiST). They are available online in the LiST Vizualiser (https://listvisualizer·org).  ^§^Odds ratios were converted into relative risks (see. Supplemental Material N°5).  ^‡^Due to lack of data available, the authors decided to perform the meta-analysis of unadjusted data.  ^†^The relative risks (RRs) presented in the pooled analysis are not adjusted. Initially, the study performed adjusted analyses on 13 of the 20 datasets. However, because multivariate adjustment did not substantially modify the estimates of associations, the researchers decided to use the crude RRs and 95% confidence intervals (CIs) for the pooled analysis. | | | | | |

1. Description of the methodology used to convert ORs to RRs

In the meta-analyses conducted by Nesari and colleagues^3^ and Vats and colleagues^6^, risk relationships were reported as odds ratios (ORs). However, relative risks (RRs) are required to estimate the population attributable fraction (PAF). Therefore, we converted the ORs from these meta-analyses into RRs using the formula developed by Zhang and Yu^7^:

$$RR=\frac{OR}{\left[ \left( 1-P_{0} \right)+\left( P_{0}\times OR \right) \right]}$$

Where RR is the relative risk, OR is the odds ratio, and P_0_ is the prevalence of the outcome of interest in the non-exposed group.

During the conversion process, we faced the challenge of not having direct access to P_0_, as data were derived from a meta-analysis. To address this issue, we retrieved P_0_ and the sample size of each original study included in the meta-analyses. We then calculated the sample size-weighted average to approximate P_0_, thereby enabling the conversion of odds ratios to relative risks for PAF estimation.

The meta-analysis by Nesari and colleagues explored the relationship between intimate partner violence before pregnancy and preterm birth.^3^ The average weighted baseline risk (​P_0_) of preterm birth in the non-exposed group was calculated to be 8·8%, derived from four studies included in the meta-analysis. The reported ORs were 1·28 (95% CI: 1·09-1·49). After converting the ORs to relative risks (RRs) using Zhang and Yu's method, the RRs were found to be 1·25 (95% CI: 1·08-1·43).

The meta-analysis by Vats and colleagues explored the relationship between pre-pregnancy overweight and obesity and stillbirth. The average weighted baseline risk (​P_0_) of stillbirth in the non-exposed group was calculated to be 0·4%, derived from eleven of the fifteen studies included in the meta-analysis for pre-pregnancy overweight and thirteen of the eighteen studies included in the meta-analysis for pre-pregnancy obesity. The reported ORs were 1·23 (95% CI: 1·12-1·36) for pre-pregnancy overweight and 1·54 (95% CI: 1·35-1·75) for pre-pregnancy obesity. After converting the ORs to relative risks (RRs), the RRs for pre-pregnancy obesity were found to be 1·54 (95% CI: 1·35-1·74) and the RRs for pre-pregnancy overweight remained equivalent to the ORs.

1. Description of the methodology used to estimate the PAF of perinatal mortality for the selected preconception risk factors

We replicated the multiple-step methodology described by Bryce and colleagues^8^ to estimate the PAFs. This methodology is particularly suited for assessing the effects of multiple risk factors simultaneously. Since women can be exposed to more than one risk factor (e.g. adolescent pregnancy and intimate partner violence), accounting for multiple effects allows for a more accurate and comprehensive understanding of the cumulative impact of these preconception risk factors on maternal and child health outcomes. The first step consisted of applying Levin’s formula to calculate the independent PAF for each risk factor:

$$\mathrm{PAF}_{u}=\frac{P_{e}\times\left( RR-1 \right)}{P_{e}\times\left( RR-1 \right)+1}$$

Where PAF_u_ is the independent PAF, P_e_ is the prevalence of the risk factor, and RR represents the relative risk of a health outcome in the population exposed to the risk factor compared to those not exposed·

Subsequently, we estimated the PAF of several preconception risk factors on the same health outcome. To do so, we applied the following formula that adjusts for multiple risk factors and produces a combined PAF:

$$\mathrm{PAF}_{t}=1-\prod_{u=1}^{n} (1-\mathrm{PAF}_{u})$$

Where PAF_t_ is the combined PAF, and PAF_u_ is the independent PAF.

To accurately reflect the proportional impact of each risk factor within the context of multiple exposures, we normalised the independent PAFs· This normalisation process results in an adjusted PAF, offering a more precise representation of each risk factor's impact on the outcome of interest:

$$\mathrm{PAF}_{a}= \left( \mathrm{PAF}_{u}/ \sum_{u=1}^{n} \mathrm{PAF}_{u} \right)\times\mathrm{PAF}_{t}$$

Where PAF_a_ is the adjusted PAF, PAF_u_ is the independent PAF, and PAF_t_ is the combined PAF.

Finally, we determined the number of health outcomes attributable to each preconception risk factor by multiplying the adjusted PAF with the total number of health outcomes in the population. To estimate the global PAFs, we aggregated the attributable health outcomes from the fifteen countries and divided this sum by the total number of observed health outcomes across these countries. This calculation yielded global PAFs that reflect the overarching contribution of the five preconception risk factors to the selected outcomes across the fifteen countries.

1. Main parameters used to estimate the economic burden associated with perinatal mortality

|  | **GDP (PPP, billion $INT)** | **Population 15-64 (per 1,000)** | **GDP per worker, $INT)** | **Retirement age** | **Minimum working age** | **LFPR, both sexes (+15)** | **Unemployment rate, both sexes** | **GNI per capita, PPP ($INT)** | **Life expectancy at median age, both sexes** | **VSL ($INT)** | **VSLY ($INT)** |
| --- | --- | --- | --- | --- | --- | --- | --- | --- | --- | --- | --- |
| **AFG** | 81 | 20,957 | 3,857 | 64 | 15 | 66·7% | 10·5% | 2,590 | 52.4 | 332,839 | 6,352 |
| **BDI** | 9 | 6,209 | 1,476 | 64 | 15 | 78·6% | 1·1% | 820 | 54.0 | 93,605 | 1,733 |
| **BEN** | 42 | 6,852 | 6,196 | 64 | 15 | 68·5% | 1·9% | 3,320 | 55.9 | 437,703 | 7,830 |
| **CIV** | 143 | 14,875 | 9,592 | 64 | 15 | 72·2% | 1·9% | 5,800 | 54.7 | 809,889 | 14,806 |
| **GIN** | 36 | 7,197 | 5,025 | 64 | 15 | 63·6% | 5·8% | 3,050 | 53.6 | 398,609 | 7,437 |
| **GMB** | 6 | 1,380 | 4,015 | 64 | 15 | 65·2% | 5·0% | 2,440 | 54.6 | 311,642 | 5,708 |
| **KEN** | 246 | 30,268 | 8,142 | 64 | 15 | 75·2% | 4·8% | 4,730 | 54.7 | 646,750 | 11,824 |
| **LBR** | 7 | 2,807 | 2,590 | 64 | 15 | 80·6% | 6·2% | 1,530 | 55.9 | 186,243 | 3,332 |
| **LSO** | 5 | 1,388 | 3,929 | 64 | 15 | 71·5% | 15·1% | 3,030 | 36.5 | 395,727 | 10,842 |
| **MLI** | 48 | 10,590 | 4,485 | 64 | 15 | 80·9% | 3·5% | 2,250 | 55.6 | 284,985 | 5,126 |
| **MRT** | 25 | 2,444 | 10,151 | 64 | 15 | 56·5% | 8·0% | 5,800 | 59.2 | 809,889 | 13,861 |
| **NIG** | 1,068 | 111,446 | 9,584 | 64 | 15 | 65·0% | 9·3% | 5,440 | 61.5 | 754,623 | 12,270 |
| **PAK** | 1,209 | 133,032 | 9,087 | 64 | 15 | 79·6% | 4·4% | 5,130 | 55.8 | 707,333 | 12,676 |
| **SLE** | 14 | 4,699 | 2,929 | 64 | 15 | 55·6% | 4·3% | 1,560 | 54.4 | 190,275 | 3,498 |
| **TCD** | 37 | 8,347 | 4,439 | 64 | 15 | 70·7% | 1·4% | 1,600 | 58.1 | 195,663 | 3,368 |
| AFG: Afghanistan, BDI: Burundi, BEN: Benin, CIV: Côte d’Ivoire, GIN: Guinea, KEN: Kenya, LBR: Liberia, LSO: Lesotho, MLI: Mali, MRT: Mauritania, NIG: Nigeria, PAK: Pakistan, SLE: Sierra Leone, TCD: Tchad.  PPP: Purchase parity power, GDP: Gross domestic product, LFPR: Labour force participation rate, GNI: Gross national income, VSL: Value of a statistical life, VSLY: Value of a statistical life-year | | | | | | | | | | | |

References

1. Kozuki N, Lee AC, Silveira MF, et al. The associations of parity and maternal age with small-for-gestational-age, preterm, and neonatal and infant mortality: a meta-analysis. *BMC Public Health* 2013; **13 Suppl 3**(Suppl 3): S2.

2. Kozuki N, Lee AC, Silveira MF, et al. The associations of birth intervals with small-for-gestational-age, preterm, and neonatal and infant mortality: a meta-analysis. *BMC Public Health* 2013; **13 Suppl 3**(Suppl 3): S3.

3. Nesari M, Olson JK, Vandermeer B, Slater L, Olson DM. Does a maternal history of abuse before pregnancy affect pregnancy outcomes? A systematic review with meta-analysis. *BMC Pregnancy Childbirth* 2018; **18**(1): 404.

4. Katz J, Lee AC, Kozuki N, et al. Mortality risk in preterm and small-for-gestational-age infants in low-income and middle-income countries: a pooled country analysis. *Lancet* 2013; **382**(9890): 417-25.

5. Female genital mutilation and obstetric outcome: WHO collaborative prospective study in six African countries. *The Lancet* 2006; **367**(9525): 1835-41.

6. Vats H, Saxena R, Sachdeva MP, Walia GK, Gupta V. Impact of maternal pre-pregnancy body mass index on maternal, fetal and neonatal adverse outcomes in the worldwide populations: A systematic review and meta-analysis. *Obes Res Clin Pract* 2021; **15**(6): 536-45.

7. Zhang J, Yu KF. What's the relative risk? A method of correcting the odds ratio in cohort studies of common outcomes. *Jama* 1998; **280**(19): 1690-1.

8. Bryce E, Gurung S, Tong H, et al. Population attributable fractions for risk factors for spontaneous preterm births in 81 low- and middle-income countries: A systematic analysis. *J Glob Health* 2022; **12**: 04013.
